# A Novel Improvement of Feature Selection for Dynamic Hand Gesture Identification Based on Double Machine Learning †

**DOI:** 10.3390/s25041126

**Published:** 2025-02-13

**Authors:** Keyue Yan, Chi-Fai Lam, Simon Fong, João Alexandre Lobo Marques, Richard Charles Millham, Sabah Mohammed

**Affiliations:** 1 Department of Computer and Information Science, University of Macau, Macau SAR 999078, China; mb85415@um.edu.mo; 2 Laboratory of Applied Neurosciences, University of Saint Joseph, Macau SAR 999078, China; alexandre.lobo@usj.edu.mo; 3 Faculty of Accounting and Informatics, Durban University of Technology, Durban 4000, South Africa; richardm1@dut.ac.za; 4 Department of Computer Science, Lakehead University, Thunder Bay, ON P7B 5E1, Canada; sabah.mohammed@lakeheadu.ca

**Keywords:** leap motion controller, dynamic hand gestures identification, feature selection, causal effect, double machine learning

## Abstract

Causal machine learning is an approach that combines causal inference and machine learning to understand and utilize causal relationships in data. In current research and applications, traditional machine learning and deep learning models always focus on prediction and pattern recognition. In contrast, causal machine learning goes a step further by revealing causal relationships between different variables. We explore a novel concept called Double Machine Learning that embraces causal machine learning in this research. The core goal is to select independent variables from a gesture identification problem that are causally related to final gesture results. This selection allows us to classify and analyze gestures more efficiently, thereby improving models’ performance and interpretability. Compared to commonly used feature selection methods such as Variance Threshold, Select From Model, Principal Component Analysis, Least Absolute Shrinkage and Selection Operator, Artificial Neural Network, and TabNet, Double Machine Learning methods focus more on causal relationships between variables rather than correlations. Our research shows that variables selected using the Double Machine Learning method perform well under different classification models, with final results significantly better than those of traditional methods. This novel Double Machine Learning-based approach offers researchers a valuable perspective for feature selection and model construction. It enhances the model’s ability to uncover causal relationships within complex data. Variables with causal significance can be more informative than those with only correlative significance, thus improving overall prediction performance and reliability.

## 1. Introduction

In recent years, with the rapid development of computer and internet technologies, Human–Computer Interaction (HCI) technologies have received wide attention. Nowadays, more and more devices are becoming intelligent and capable of interacting with humans, driving the evolution of user interaction. Traditionally, most systems relied on keyboards, mice, and touchscreens as input devices for HCI. However, with the emergence of diverse technologies such as the Internet of Things (IoT), the development of more human-oriented interaction methods has become particularly important. The core of HCI technology lies in designing, implementing, and evaluating interactive interfaces to enhance the user’s experience with computing devices. Traditional touch-based interaction devices, such as keyboards and touchscreens, have revealed some shortcomings. Especially during the COVID-19 epidemic period, most of users’ concerns for health and hygiene have increased significantly, and many prefer to perform operations without physical contact [1,2]. For example, technologies such as voice recognition and gesture control manage to make HCI more natural and efficient. These emerging technologies can not only meet users’ needs for convenience and security, but also provide a richer and more immersive interaction experience.

Hands are the most frequently used part of the human body. Humans can use their hands to make a variety of gestures and define simple combinations of gestures to perform complex commands. Flexible, natural gestures have greater versatility than traditional touch methods [3]. In general, gesture identification can be divided into two main categories. The first category is computer vision-based recognition techniques, which capture hand images through one or more cameras and utilize deep learning algorithms, such as neural networks and convolutional neural networks, to classify gestures [4]. However, vision-based methods require image processing to extract hand position information, making them costly, especially for dynamic gesture identification. The second category is sensor-based recognition techniques, such as using digital gloves and 3D depth sensors to collect and analyze data. Although sensor-based methods have been around since the 1970s, their practical application has been limited because they always rely on wearable devices, such as digital gloves, for tracking and estimating the position and orientation of the hand and fingers [5]. With the development of 3D depth sensor technology, tools like the Leap Motion Controller (LMC) have emerged to provide a low-cost and more efficient solution for detecting and tracking hand motion. The LMC can output data such as palm orientation, fingertip position, bone position, and other relevant points.

With the abundance of LMC data types, the number of variables available for research is increasing. Feature selection is particularly important when dealing with high-dimensional data, as it can significantly reduce the time cost and model complexity in the analysis process. In recent years, research on feature selection has gained attention, with statistical and optimization-based methods such as Recursive Feature Elimination, Chi-Square, and Genetic Algorithm commonly used to reduce data dimensionality and improve the efficiency of model training [6]. Additionally, causal machine learning leverages the strong fitting ability of machine learning and deep learning models to characterize the positive or negative impact of individual variables on the final dependent variable outcome by observing changes in the treatment effect value [7]. In this research, we propose a novel feature selection method using the Double Machine Learning (DML) model. By controlling for other variables, the DML model can assess whether there is a significant difference in the treatment effect of a single variable on the dependent variable. This research extends the findings of our conference paper [8]. In comparison to the original paper, we incorporate additional feature selection methods and machine learning models for a comprehensive comparative study. For each dataset, all feature selection methods are tuned with parameters similar to those used in the DML method. Consequently, this research provides a more rigorous verification that DML is more effective for feature extraction in gesture identification recognition problems.

The remainder of this paper is organized as follows: Section 2 reviews LMC-based classification papers, as well as the research related to machine learning and deep learning, and explores the application of feature selection and DML. Section 3 describes the experimental environment setup, datasets, feature selection methods, and machine learning methods in detail. Section 4 presents the detailed experimental procedure and results. Finally, Section 5 summarizes the research.

## 2. Related Works

The LMC is an advanced gesture identification device that has been the subject of many significant research papers in the fields of gesture identification and human–computer interaction. It is capable of outputting data such as palm orientation, fingertip position, bone position, and other relevant points. Its high Accuracy and real-time capabilities make it promising for a wide range of applications across several fields. Weichert et al. analyzed the Accuracy and robustness of the LMC in detail, finding that it had minimal deviation in both static and dynamic settings of fingertip position, especially in static settings where deviation is minimized [9]. In the field of static gesture identification, Marin et al. conducted a study using three feature datasets (fingertip distance, fingertip angle, and fingertip height) collected by the LMC. They found that the Accuracy of static gesture identification based on the LMC could reach 80% [10]. This result indicated that the LMC had high reliability and Accuracy in static gesture identification, with the potential to meet the needs of real-world applications in the future. The application of the LMC in virtual reality and augmented reality has also gained significant attention. The LMC can provide high-precision hand tracking, enabling game users to interact naturally in virtual environments. Martins et al. evaluated the effectiveness of the LMC in a 2D gaming environment, and found that it performed better in the collection of game items and during combat, but not as well as traditional mouse and keyboard in character movement [11]. The LMC has also shown great potential for applications in the medical and rehabilitation fields due to its contactless interaction characteristics. Ameur et al. defined 11 dynamic gestures for medical image manipulation to address the need for aseptic manipulation in healthcare, using 3D positional information as an input feature for the model [12]. This approach not only improves the ease of operation, but also effectively reduces the risk of cross-infection.

In terms of dataset expansion and application, researchers continue to expand and enrich datasets to further improve the Accuracy of gesture identification. A research team released a dataset containing more samples and richer spatial-frequency features [13], providing a valuable resource for subsequent studies and helping to enhance the performance of gesture identification models. The LMC is able to provide detailed data on skeletal movements. Boulahia et al. extended the existing action recognition feature set HIF3D, and collected a new dataset containing 46 joints (in 3D coordinates) using the LMC [14]. These dataset includes information on the 3D coordinates of 23 joints of each hand, offering researchers more detailed skeleton data and improving the representation of whole-body gestures. In terms of skeleton gesture identification, Li et al. proposed a skeleton-based gesture identification enhancement method [15]. Their results showed that the Accuracy of static gesture identification was as high as 94%, while the dynamic gesture identification rate, combined with the initial FC strategy, exceeded 90%, which provided new ideas and directions for the development of gesture identification technology. More summaries of gesture datasets can be found in Chakravarthi et al. These datasets covered various types of gestures and specific application scenarios, providing meaningful references for researchers [16].

With the increasing richness of LMC data types, the number of variables available for categorization has gradually increased. Feature engineering is particularly important when dealing with high-dimensional data, as it can significantly reduce the time cost and complexity of the classification process. In recent years, research on feature engineering has become popular, and many statistically based methods have been widely used for feature selection, such as Recursive Feature Elimination, Chi-Square, and Genetic Algorithm [6]. These methods improve the efficiency of model training by reducing data dimensionality. Among them, Recursive Feature Elimination is a commonly used method that selects features by recursively constructing the model and eliminating the least important features. The Chi-Square test evaluates the correlation between categorical variables to select the most relevant features. The Genetic Algorithm is an optimization algorithm based on the principles of natural selection and genetics, which selects the optimal subset of features by simulating the process of biological evolution [17]. In addition to the problem of data complexity, the complexity of machine learning models is increasing as models continue to evolve, making the interpretability of models increasingly important in different research areas [18]. Interpretable machine learning aims to make the decision-making process of a model transparent and help users understand the model’s behavior and the basis of its decisions. Shapley Additive Explanations (SHAP) is a commonly used interpretable method in machine learning modeling, explaining the model’s decisions by calculating the value of each feature’s contribution to the model’s output [19]. However, in machine learning tasks, the model’s understanding of the relationship between independent and dependent variables during training is based on correlation instead of causality. It means the model tends to focus more on the correlation between dependent and independent variables when identifying variable importance [20]. Causal machine learning, on the other hand, combines the strengths of causal inference and machine learning to improve the interpretability and predictive power of the model by modeling the causal relationships between variables. It utilizes the strong fit of machine learning models to describe the positive or negative impact of individual variables on the outcome of the final dependent variable through changes in treatment effect values [7]. This approach not only improves the predictive Accuracy of the model, but also provides deeper insights to help understand the causal relationships between variables. Currently, causal uplift modeling has applications in business, healthcare, and other industries [21]. Among these, Wijaya et al. quantified the effect of different dependent variables on employee turnover classification using uplift modeling from a causal inference perspective [22]. One research further explored this research methodology and demonstrated the application of the uplift modeling to interpretable machine learning [23]. The uplift modeling not only provides finer causal inference at the individual level, but also complements the SHAP diagram to offer a more comprehensive explanation of the variables.

## 3. Materials and Methods

This section explores the data collection process, the construction of variables, the methods of feature selection, and the construction of machine learning models in detail. First, we introduce the specific steps of data collection, the environment, and the variables involved to ensure the completeness and Accuracy of the data. Next, we provide an in-depth introduction to the DML-based novel feature extraction method used in this research, describing its principles and application scenarios, as well as the feature selection method used for comparative study with DML. Finally, we outline several major machine learning classification methods used in the experiments, the evaluation metrics, and discuss their applicability and performance in this research.

### 3.1. Gesture Datasets Collection

The LMC from Ultraleap is an optical hand-tracking module that captures the movement of users’ hands and fingers, allowing them to interact naturally with digital content. The LMC includes three IR LEDs for scene illumination and two CMOS cameras that capture images at frame rates ranging from 50 to 200 frames per second. It can track hands and fingers within a 3D interactive zone extending up to 60 cm or more from the device, with a typical field of view of 140 × 120° [24].

**Figure 1:**
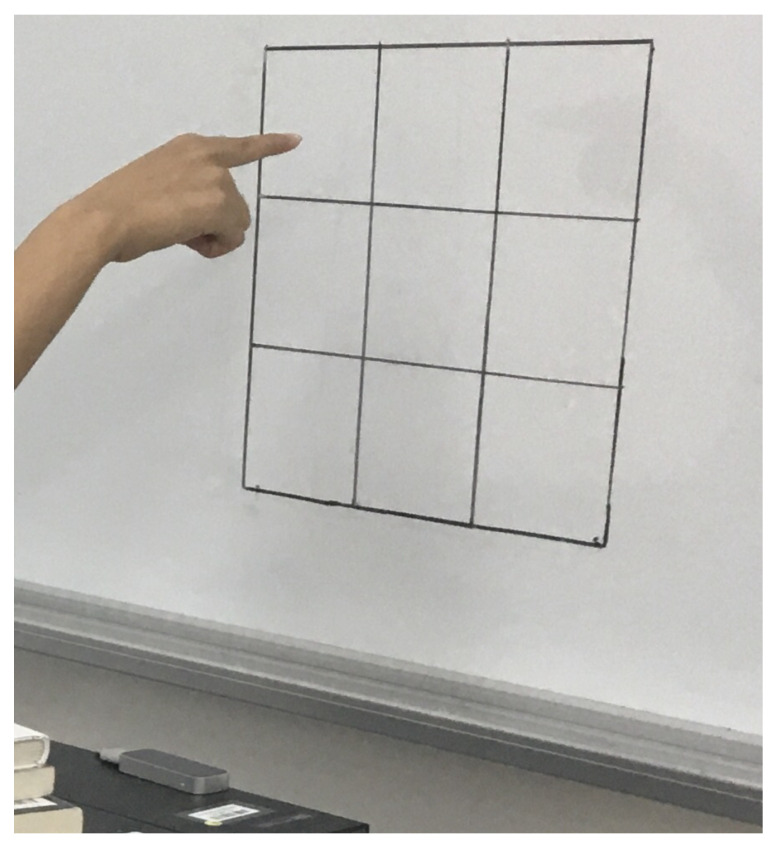
Initial step of collecting data.

We collected all datasets in a controlled indoor environment to ensure consistency of experimental conditions and reliability of data. The LMC device was placed on a flat tabletop in front of a whiteboard. Depending on the extent of the data collected by the LMC, we drew a 3 × 3 square grid on the whiteboard with a marker pen, with each grid measuring 10 × 10 cm. All experimental participants made gestures 20–30 cm above the LMC device to ensure it could capture the complete gesture movement trajectory. The LMC was connected to a laptop computer via a USB cable, and a researcher monitored and controlled the entire data collection process to ensure the Accuracy and integrity of the data. During the experiment, all participants were asked to place their hands on the LMC and point their fingers at the whiteboard as the initial hand position. The data collection process is shown in Figure 1.

**Figure 2:**
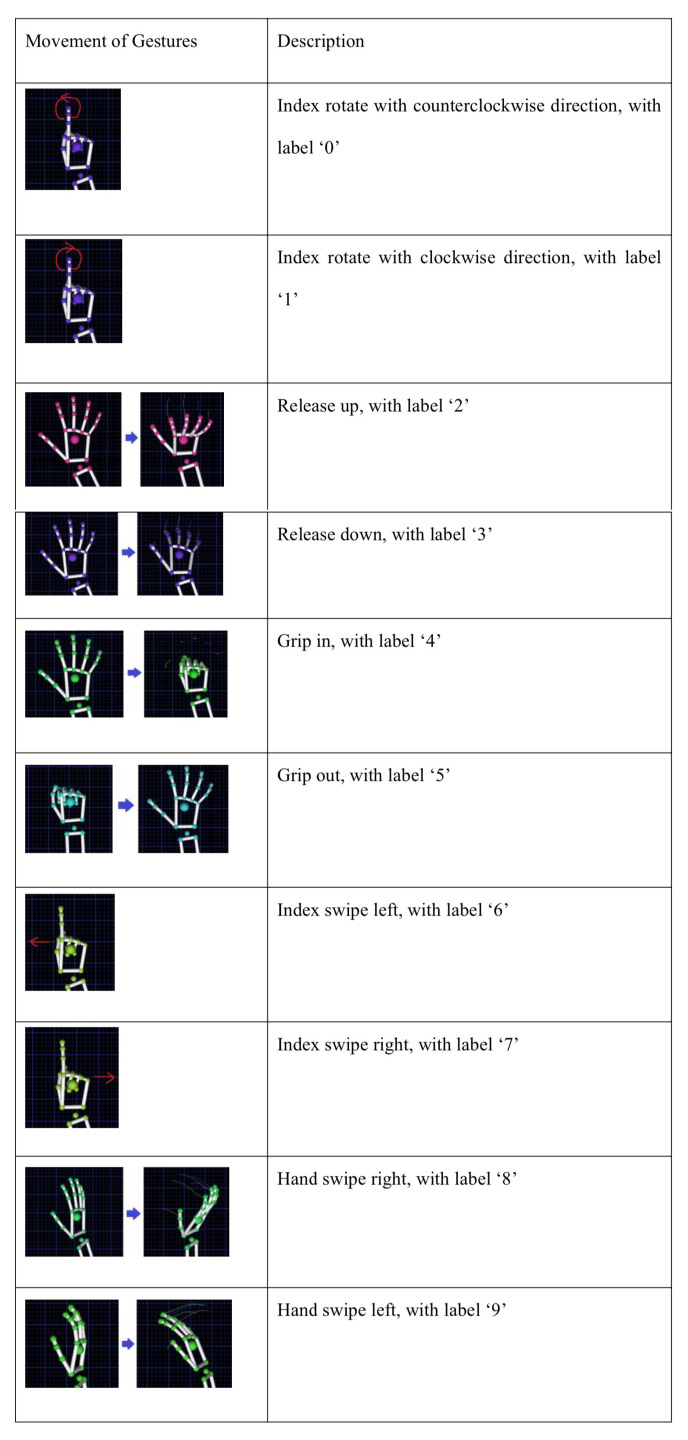
Description of hand gestures.

A total of 12 participants, including 7 females and 5 males, were invited to the experiment. To ensure diversity and representability of the data, the participants were instructed to perform a series of predefined hand movements. The experimental design consisted of 10 different movements, with each participant repeating each movement 10 times. Specific movement types and descriptions are detailed in Figure 2. The entire experimental process was conducted in strict accordance with predefined steps to ensure the repeatable and scientific validity of the data. The researcher monitored the participants’ movements closely during the data collection process to ensure that each movement was recorded accurately.

### 3.2. Structure of Gesture Datasets

To process time series motion data collected by LMC, we propose using the temporal windowing technique to associate several frames. Thus, we converted the hand motion frame data into a feature set based on the number of time windows. While recording the hand gesture data for all participants, we notice that the time windows significantly affected the classification. Considering the calculation of standard deviation, the number of time windows is limited to 35. By comparing the data with window numbers of 5, 10, 15, 20, 25, 30, and 35, we find that when the number of windows is 15, the initial computed classification Accuracies are better and models run faster. Specifically, we calculate the arithmetic mean and standard deviation for each time window to construct a unique set of features. Suppose the feature set is $W_{i}=\left\{f_{1},f_{2},f_{3},\ldots ,f_{m-1},f_{m}\right\}$ , where *m* denotes the number of features. All location data in the feature set is normalized to the range of [−1, 1]. In this research, 15 time windows are used, i.e., i=15 . All variables are combined as $\left\{W_{1},W_{2},\ldots ,W_{14},W_{15}\right\}$ .

We utilize three types of feature sets: single finger features, two finger features, and bone finger features. To provide information about the direction of the action, we add finger direction, palm direction, and palm normal vector to all feature sets. The single finger feature set focuses on the 3D spatial features of the hand and fingers. The variables involved in the single finger features are hand position, hand direction and normal, fingertips position, and fingertips direction. We divide them into two datasets: single finger feature mean (fset_mean), which contains the mean of the variables, and single finger feature standard deviation (fset_std), which contains the standard deviation of the variables. The formulas for calculating the mean and standard deviation are shown below:

(1) $$Mean\left(V\right)=\frac{1}{n}\sum_{i=1}^{n}V_{i}$$

(2) $$Stdev\left(V\right)=\sqrt{\frac{1}{n}\sum_{i=1}^{n}\left(V_{i}-Mean\left(V\right)\right)}$$

In the two finger feature set (fset_dist), we include fingertip palm distances, adjacent fingertip distances, hand direction and normal, and fingertips direction. These features help evaluate the performance of distance-based gesture identification, especially in complex gesture identification tasks. The bone finger feature set (fset_bones_mean) contains the positions of the distal and middle phalanges as 3D spatial features of the bone finger. Specific variables include hand position, hand direction and normal, fingertips position, fingertips direction, distal phalanges position, and intermediate phalanges position. These features provide more detailed skeletal information for gesture identification and help improve the Accuracy of identification. The main features involved in the four datasets are shown in Table 1.

### 3.3. Feature Selection Methods

In the information age, feature selection plays a crucial role in the data preprocessing process before machine learning and data mining. By identifying and selecting the most informative features, feature selection not only improves the performance of the model significantly, but also reduces computational complexity and the risk of overfitting. In practice, there are various feature selection methods, including Variance Threshold (VAR), Select From Model (SFM), Principal Component Analysis (PCA), and Least Absolute Shrinkage and Selection Operator (LASSO), while current deep learning-based selection methods include Artificial Neural Network (ANN) and TabNet.

The VAR method is a simple but effective approach for feature selection. It measures the dispersion of features by calculating the variance value of each feature. The smaller the variance, the less information the feature provides. Therefore, we can set a variance threshold and eliminate features that fall below this threshold to achieve feature selection [25]. The advantage of this method is simplicity and suitability for initial feature screening, but the disadvantage is that it fails to consider the correlation between features. As another traditional method, SFM is a more complex and precise feature selection. It evaluates the importance of features by using machine learning to analyze the parameters or variable importance of the model to decide which features should be retained. Among these, Random Forest is often used as a machine learning model for selecting variables. The Random Forest model evaluates the importance of each feature by constructing multiple decision trees and combining their predictions. This approach considers both the individual contributions of the features and the interactions between them, making it very effective in practical applications. PCA is a feature selection technique that differs from the previous methods. The core of PCA is to compress feature dimensions rather than selecting features. PCA projects high-dimensional data into a low-dimensional space, preserving the main information of all features and simplifying the data structure. Specifically, PCA transforms the original features into a new set of uncorrelated features through linear transformation, called principal components. These principal components are ranked according to the magnitude of their explained variance, and the first few principal components usually contain most of the data’s information, allowing them to replace the original features [26]. LASSO is a feature selection method commonly used in statistics. LASSO introduces an L1 regularization term in the regression model, causing some regression coefficients to zero, thereby excluding variables with zero coefficients. This method not only improves the predictive performance of the model, but also enhances its interpretability. Due to its simplicity and efficiency, LASSO is widely used in machine learning research, particularly in the medical field [27,28].

As deep learning becomes popular, this research employs the classical ANN model for feature selection. The principle is to select features based on the neuron weights of the ANN. By analyzing the weights of neurons in each layer at the end of ANN training, we identify and select the most important features for the model output. The core idea of this method is to leverage the adaptive learning ability of neural networks to adjust weights through the backward propagation algorithm, thereby automatically screening the features that have the most impact on prediction results. Some papers have examined the effectiveness of ANN in feature selection and compared it with different feature selection methods [29,30]. Similarly, there is a feature selection method based on the neuron weights of TabNet, which achieves feature selection through the Sequential Attention Mechanism. This method is currently applied in various fields [31,32].

In traditional causal learning for the effect of treatment, researchers often focus on the effect of heterogeneity by dividing the sample into control and experimental groups and calculating the significant difference in effect when other variables are similar. The randomized controlled trial is recognized as the gold standard for this purpose [33]. However, due to ethical considerations, especially in medical area, researchers often need to calculate the treatment effect based on existing data sets. Some well-known methods such as Differences-in-Differences and Propensity Score Matching have been used in many aspects [34,35]. Now, with the development of machine learning, more causal models have been invented and used. For discrete datasets, S Learner, T Learner, and X Learner can be used to test for heterogeneity. For data with continuous variables, we can use DML to analyze them [36]. In this paper, we propose an innovative approach for feature selection based on DML. It assumes that all potential factors or variables are defined as observed confounder *X*. *T* stands for Treatment, while outcome *Y* denotes the affected dependent variable. In practice, the confounding variable *X* can be challenging to model satisfactorily due to its high dimensionality. The DML approach creates a model capable of capturing heterogeneous treatment effects by combining two machine learning predictive models into a final stage estimation. This approach allows for the usage of arbitrary machine learning algorithms for both prediction tasks, thereby increasing the model’s flexibility and adaptability [37,38]. The DML approach involves two separate predictions in two separate machine learning models for the treatment variable *T* and the outcome *Y* in the first stage. In the second stage, these predictions are considered as new features that are used as the final causal effect estimation model.

**Figure 3:**
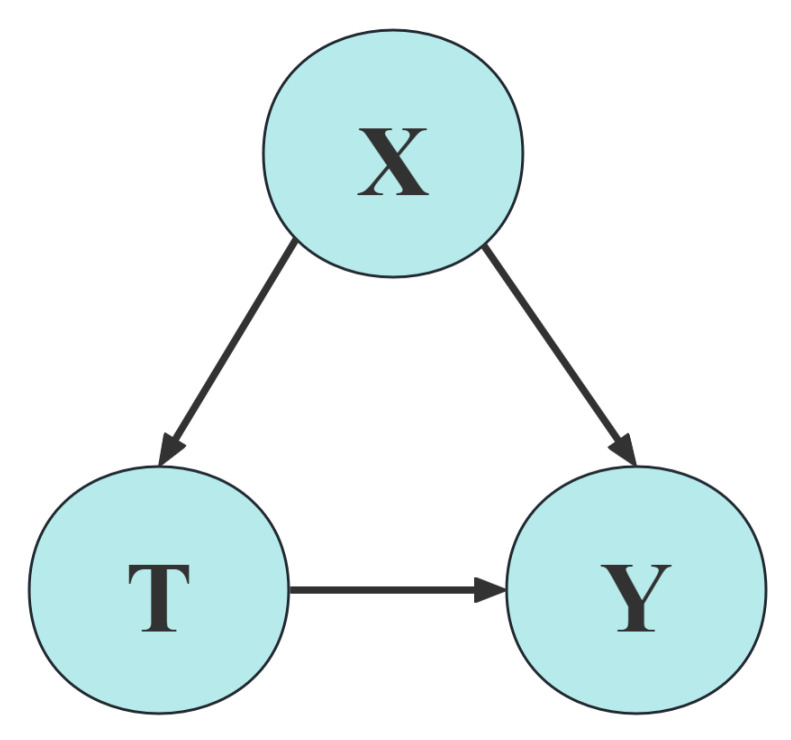
Structure of *X*, *T* and *Y*.

As shown in Figure 3, the variables *T* and *X* affect the dependent variable *Y* together, while *X* also influences *T*. In the DML approach, we consider both *Y* and *T* as dependent variables, and compute the differential effect on *Y* at different values of *T* by estimating the effect of features on these two dependent variables. This method allows us to more accurately capture the effects of the high dimensional confounding variable *X* on *Y* and *T*, thereby improving the Accuracy and robustness of the causal effect estimation. These formulas are shown below:

(3) $$E\left(Y|X\right)=ML_{1}\left(X\right)$$

(4) $$E\left(T|X\right)=ML_{2}\left(X\right)$$

We can then calculate the residuals of the variables *Y* and *T*, and perform the corresponding regression analysis to obtain the coefficient θ , also known as the Conditional Average Treatment Effect (CATE). The CATE provides a precise measure of the heterogeneity of the effect across all samples, leading to a better understanding of how different characteristics influence the mechanisms affecting the dependent variable.

(5) $$Y-ML_{1}\left(X\right)=\theta \left[T-ML_{1}\left(X\right)\right]+\epsilon$$

When the CATE shows a significant positive or negative difference at a 90% confidence interval, we can confirm that the variable *T* has a significant effect on the outcome *Y*. In this research, we treat each of the independent variables in the four datasets individually as a treatment variable *T* and the remaining independent variables as confounding variables *X*. By training the DML model, we test whether each variable has a significant positive or negative difference in CATE. For those independent variables with significant differences, we select them as key features for the next machine learning classification task. To verify the feasibility of the DML-based feature selection method, we compare it with several other feature selection methods mentioned earlier. By comparing the performance of different methods in various classification tasks, we can assess the effectiveness and advantages of DML methods in feature selection.

### 3.4. Machine Learning Models

For the selection of machine learning models, we choose six classical and widely used models for analysis: Logistic Regression (LR), K-Nearest Neighbors (KNN), Random Forest (RF), Extra Trees (ET), Histogram-based Gradient Boosting (Hist-GB), and Light Gradient Boosting Machine (LightGBM). These traditional models have broad applications and perform well in various fields, handling different types of data and tasks [39].

For deep learning models, we select three: ANN, 1-Dimensional Convolutional Neural Network (1D-CNN), and TabNet. ANN is simple in structure, easy to implement [40]. The 1-Dimensional CNN, a variant of CNN, is specifically designed to process one-dimensional data such as time series and signals. It extracts local features from the data through convolutional operations and reduces feature dimensions through a pooling layer, improving the model’s efficiency and generalization [41]. TabNet is a deep learning model for tabular data that uses the sequential attention mechanism for feature selection and data processing. Arik and Pfister first proposed TabNet in their seminal study, demonstrating its excellent performance on multiple datasets [42,43].

In our analysis, we randomly divide the dataset into training and testing sets, with 80% of the data used to train models and the remaining 20% used to test the models’ performance. To comprehensively evaluate the classification effectiveness of models, we adopt four commonly used classification metrics: Accuracy, Precision, Recall, and F1-Score. These metrics measure the models’ performance from different perspectives,

(6) $$\mathrm{Accuracy}=\frac{TP+TN}{TP+TN+FP+FN}$$

(7) $$\mathrm{Precision}=\frac{TP}{TP+FP}$$

(8) $$\mathrm{Recall}=\frac{TP}{TP+FN}$$

(9) $$F1\text{-}\mathrm{Score}=\frac{2\times TP}{2\times TP+FP+FN}=2\times \frac{\mathrm{Precision}\times \mathrm{Recall}}{\mathrm{Precision}+\mathrm{Recall}}$$

The closer the value of these indicators is to 1, the better the model’s classification performance and the smaller the error. In practical applications, we use these indicators to evaluate and compare the performance of different models, allowing us to select the most suitable model for further optimization and application.

## 4. Experience Results

**Figure 4:**
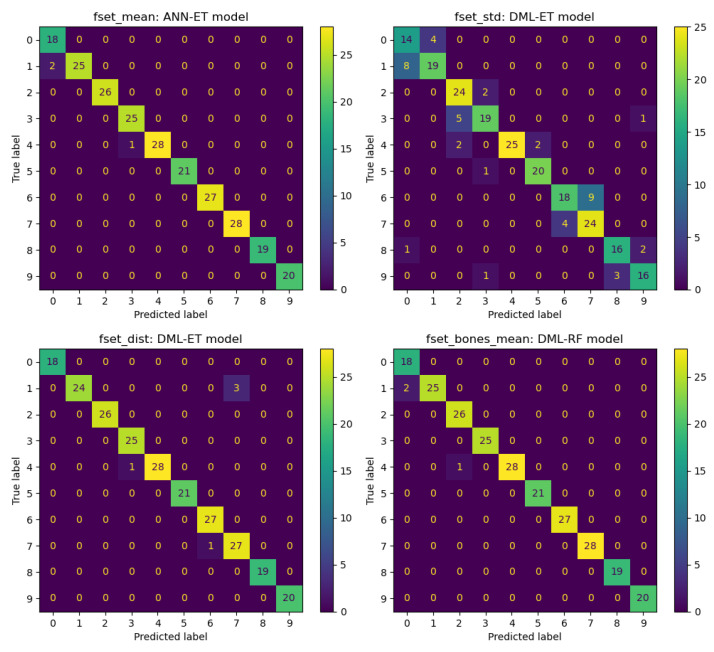
Best classification results for different datasets.

In this section, we show details of predictions obtained using various feature selection methods and machine learning algorithms. We conduct experiments on four datasets: fset_mean, fset_std, fset_dist, and fset_bones_mean. By combining seven feature selection methods with nine machine learning models, we obtain 63 combinations. First, we categorize the datasets and evaluate the performance of these 63 combinations across the four datasets: fset_mean, fset_std, fset_dist, and fset_bones_mean. Our results show that the ANN-based feature selection method combined with the ET machine learning model provides the best predictions for the fset_mean dataset. Similarly, for the fset_std dataset, the DML method combined with the ET model yields the best results. The DML-ET model also performs best on the fset_dist dataset, while the DML-RF model has the best prediction effect based on the fset_bones_mean dataset. Figure 4 presents the confusion matrix visualization plots for these four prediction results. Comparing these plots reveals that the machine learning models perform relatively well on the fset_mean, fset_dist, and fset_bones_mean datasets, with fewer classification errors. However, performance on the fset_dist dataset is relatively average, with more classification errors. For further analysis, the ANN feature selection method used in the fset_mean dataset significantly improves the ET model’s prediction Accuracy. Additionally, the DML method combined with the ET model demonstrates excellent classification ability on the fset_std and fset_dist datasets. On the fset_bones_mean dataset, the DML method combined with the RF model also shows outstanding performance, highlighting the adaptability and effectiveness of the DML feature extraction method across different datasets.

To fairly assess the classification prediction effectiveness of these combinations, we use the Mean value ± Standard Deviation (Mean ± Std) as an evaluation metric to observe the average performance of all feature selection methods and classification models across the four datasets. The results are shown in Table 2. The situation here differs slightly from Figure 4. The table shows that the Accuracy of the fset_mean and fset_bones_mean datasets is very high, with an average Accuracy exceeding 93%, regardless of the feature selection methods and machine learning models used. In contrast, the fset_dist dataset, which captures positional variations, has an average Accuracy 5–6% lower than the former two. The fset_std dataset performs the worst, with an average Accuracy of less than 70%. Further analysis of the standard deviation data in the table reveals that the fset_mean and fset_bones_mean datasets exhibit good stability, with small fluctuation ranges in Accuracy and other metrics, having a standard deviation between 5% and 6%. On the other hand, the fset_dist and fset_std datasets show more variability, with standard deviations ranging from 9% to 10%. This suggests that the fset_mean and fset_bones_mean datasets are more reliable for real-world classification applications, while the feasibility of using the fset_dist and fset_std datasets for analysis needs to be carefully considered.

**Figure 5:**
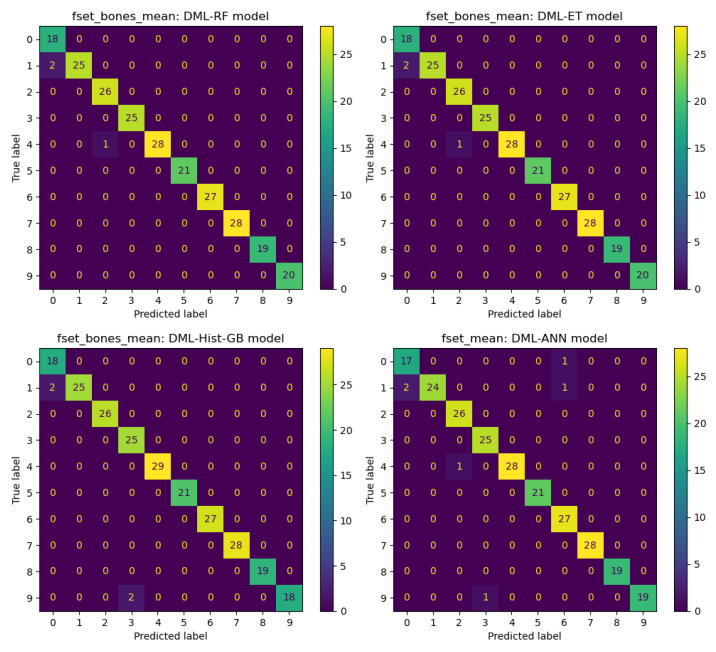
Best classification results for different machine learning models.

In the next analysis, we compute the classification results using nine machine learning models. We evaluate the performance of these models across all datasets and feature selection methods, ultimately selecting the four best-performing models: DML-RF, DML-ET, DML-Hist-GB, and DML-ANN. These four models perform best on the fset_bones_mean and fset_mean datasets, as shown in Figure 5. Similarly to the models selected in Figure 4, the DML-based feature selection method demonstrates good applicability to most machine learning models. The features selected through the DML method have a significant positive impact on predictive classification.

Individual machine learning models have inherent limitations, so we also evaluate the average performance of all machine learning models, as shown in Table 3. The LR and KNN models, which are based on single classifiers, perform the most averagely, with Accuracies around 77% and 84%, respectively. This performance is significantly lower than that of the tree-based machine learning models, which achieve Accuracies between 88% and 90%. Additionally, the prediction results of the LR and KNN models fluctuate widely, indicating lower stability. Among the four tree-based machine learning models, the ET model performs the best, delivering excellent prediction results with low volatility. Similarly, several common deep learning models, including ANN, 1D-CNN, and TabNet, are used for comparative studies. The results show that ANN achieves the best classification results, with average Accuracy comparable to that of tree-based machine learning models. The 1D-CNN model’s classification performance is only slightly better than that of the single classifier models, but its results fluctuate and are less stable. Surprisingly, the TabNet model does not perform as well, with average Accuracy only slightly better than the linear model LR and the worst stability among the nine models.

This suggests that, in terms of feature extraction, tree-based machine learning models provide better classification results, while deep learning feature extraction methods may not be suitable for this type of LMC-collected skeletal data, but may have better applications in other areas. From this set of experiments, we can conclude the following:

- The characteristics of different datasets also impact model performance.
- The choice of feature selection methods and machine learning models significantly affects classification prediction performance. DML methods and tree-based machine learning models perform well in classification prediction with high Accuracy and stability, especially the ET model.

Based on these results, we recommend prioritizing tree-based ET model in practical applications due to their robustness and stability.

**Figure 6:**
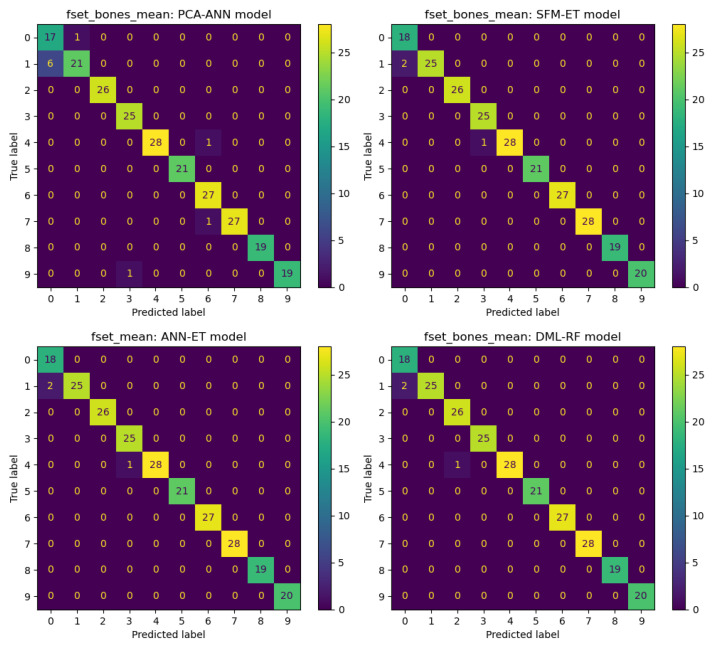
Best classification results for different feature selection methods.

Figure 6 illustrates the classification results using the four best-performing feature selection methods: PCA-ANN, SFM-ET, ANN-ET, and DML-RF models. Similarly to Figure 5, the datasets used for these top-performing models are fset_bones_mean and fset_mean. There is almost no difference in the classification results of these four models regarding the best results for each feature selection method. However, this research focuses more on the generalization and robustness of the methods. It is expected that feature selection methods manage to perform well across different datasets and machine learning models.

**Figure 7:**
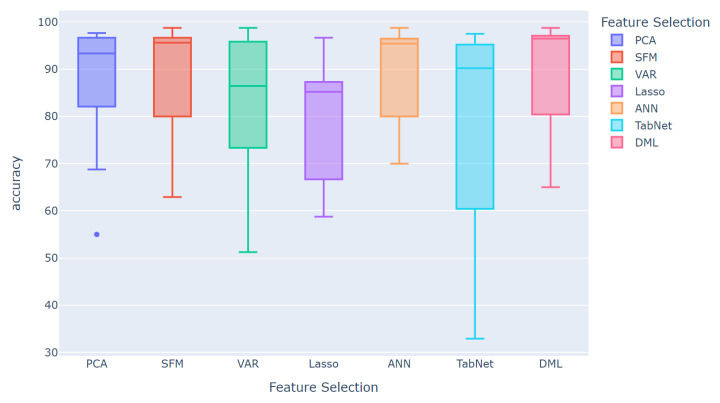
Comparisons of Accuracy based on feature selection methods.

We test the performance of all datasets and machine learning models using the seven feature selection methods. As shown in Table 4 and Figure 7, these results highlight the superiority of our proposed novel DML-based feature selection method. The average Accuracy achieved by the traditional PCA-, SFM-, VAR-, and LASSO-based feature selection methods in predicting the nine machine learning models does not exceed 90%. Among them, PCA and SFM perform the best, with Accuracies over 89%, while VAR achieves about 85%, and LASSO averages below 80%. For deep learning feature extraction methods, ANN performs better, with an average Accuracy of 89.8%, while TabNet’s performance is more comparable to the linear regression-based LASSO model. Similarly, Figure 7 shows that the overall performance of LASSO and TabNet is indeed poor, with TabNet’s performance fluctuating significantly. Table 4 and Figure 7 both show that the extraction effect of ANN is not as effective as DML. The ANN extraction method based on deep learning has the disadvantage of less interpretable features, whereas the DML method, selected based on the significance of the difference in the CATE value, offers higher interpretability and reliability in various fields. Compared to all methods, the DML method achieves an average prediction Accuracy of over 90%, the best performance among all feature extraction methods. Figure 4 and Figure 5 demonstrate that data processed by DML methods consistently achieve the highest Accuracy rates, regardless of the dataset or machine learning model used. This further proves the adaptability and effectiveness of the DML method across different datasets and models.

To observe the performance of the feature selection method across each dataset and machine learning model more intuitively, we try to conduct more in-depth analyses. The results of these analyses not only validate the superiority of the DML method, but also provide valuable references for future research.

We divide the results into the performance of classification metrics based on feature selection methods and datasets. For more convenient and effective presentation, we select the five best-performing feature selection methods from Table 4: PCA, SFM, VAR, ANN, and DML, as shown in Table 5. Similarly to the results in Table 2, we can see that all machine learning models perform very well on the fset_mean and fset_bones_mean datasets, with Accuracies exceeding 95% in most cases. The only exception is the fset_mean dataset based on the VAR method, which performs relatively average, with an Accuracy of about 91%. These two datasets contain features highly relevant to the research topic of gesture identification, leading to better performance. Moreover, the DML method achieves the best classification results in both the fset_mean and fset_bones_mean datasets. In the fset_bones_mean dataset, the DML method has an Accuracy of 96.824%, about 0.2% higher than the second-ranked VAR method. In the fset_std and fset_dist datasets, the DML method still maintains its leading position, although the prediction Accuracies of all methods under these two datasets are relatively low. These results indicate that DML methods have high reliability and adaptability across different kinds of datasets, outperforming traditional feature selection methods and deep learning-based feature extraction methods. Additionally, the DML method provides important reference value in variable interpretation.

**Figure 8:**
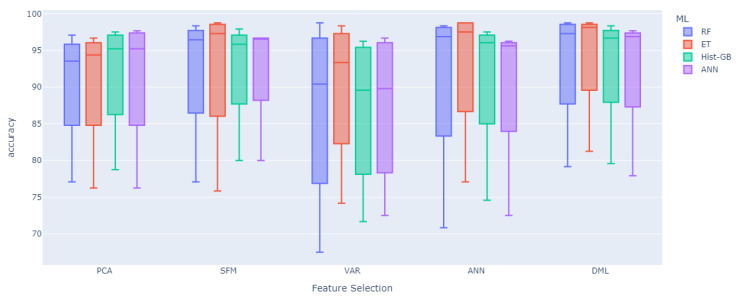
Comparisons of Accuracy based on feature selection methods and machine learning models.

Finally, we divide the results into the performance of classification metrics based on feature selection methods and machine learning models. To present these results more conveniently and effectively, we select the five best-performing feature selection methods from Table 4: PCA, SFM, VAR, ANN, and DML. Meanwhile, based on Table 4, we select the four machine learning models with the best average performance: RF, ET, Hist-GB, and ANN. The computational results are shown in Table 6 and Figure 8.

From the results in Table 6 and Figure 8, we can clearly find that the VAR-based method has the most average classification results. In all the machine learning models using the VAR feature extraction method, their classification indexes do not exceed 90%, with some results even falling between 86 and 87%. This indicates that not only does the underlying data impact the model, but the choice of feature selection method is also crucial. Among the feature selection methods, the performance of PCA and ANN is comparable, with Accuracies ranging between 90 and 92%. The gap between the upper and lower limits of ANN extraction methods can be seen in Figure 8 and, similarly, the fluctuation of ANN in Table 6 is relatively large, with a standard deviation around 10%, much higher than the other four feature extraction methods. Moreover, the ANN variable importance extraction method has the drawback of the neural network’s black-box nature, making the variables unreliable in scenarios or studies that require more interpretability. PCA, which composes new variables through linear transformation, also falls short in terms of interpretability.

The performances of the remaining SFM and DML methods are also relatively close, as shown in Figure 8, where the boxplot distributions of these two feature selection methods are similar. In the ANN classification model, DML performs slightly worse than SFM. However, in the remaining three tree-based machine learning models, DML outperforms SFM in terms of Accuracy. Notably, under the ET model, DML improves by about 2% points over the SFM method. This indicates that the DML variable extraction method has better performance, attributed to its ability to seek the causal relationship between independent and dependent variables while also selecting the variable indicators that are more important for the results. Therefore, using DML for variable selection is more advantageous.

According to the results in Table 4, Table 5 and Table 6 and Figure 8, our research find that the feature selection method and the choice of machine learning model significantly affect classification performance. PCA and ANN perform well in terms of Accuracy, but lack interpretability. SFM and DML perform well across different models, with DML performing particularly well in tree-based models. These findings provide important references for future research and applications.

## 5. Conclusions

In this research, we introduce a new feature selection method for the gesture identification task using gesture data collected by the LMC device. Building on previous research [8], we appropriately extended various methods. Overall, we employ the DML method in causal machine learning for effective selection of the dependent variable, alongside six other feature selection methods and nine machine learning algorithms, to conduct a comparative study on four different types of datasets. This approach ultimately achieves excellent classification results. Through these experiments, we have drawn the following conclusions:

- The characteristics of different datasets significantly affect model performance. The fset_mean and fset_bones_mean datasets are preferable for classification prediction due to their high Accuracy and stability. Conversely, the fset_dist and fset_std datasets should be used with caution in practical applications due to their higher volatility and lower average Accuracy.
- The choice and combination of feature selection methods and machine learning models significantly impact classification prediction effectiveness. Linear classifiers or single classifiers (e.g., LR and KNN) are generally less effective, while tree-based machine learning models perform better in such tasks, especially ET models. Additionally, DML method based on causal inference has a significant advantage in feature selection, even slightly outperforming feature extraction methods based on ANN models. DML achieves the best classification results while ensuring interpretability.

Through these analyses, we can understand the performance of different feature selection methods and machine learning models on various datasets better. This not only verifies the superiority of the DML method, but also provides a new perspective for feature engineering research. In future research, we can further explore how to optimize these combinations and extend the application of DML to different research topics and datasets. For example, we can add more diverse gestures to the dataset or validate the application of causal inference in feature engineering using other models such as Linear DML, Sparse Linear DML, and Causal Forest DML. Furthermore, when facing with other research topics or datasets, we can explore the application of causal inference methods in explainable learning and feature engineering. In the next step, we will also conduct comparative investigations of different methods using well-known financial, medical, commercial, and other public datasets to determine which causal inference methods are more reliable. These findings can help us make more informed decisions in real-world engineering projects, thereby improving overall classification prediction performance.

## Figures and Tables

**Table 1:** Main features of different datasets.

Dataset	Main Features	Description
fset_mean	m_{figure}_pos_{position}_{i}	the mean value of figure (thumb, index, middle, ring, pinky) position of (X axis, Y axis, Z axis) in 3D coordinated system with window i (0, 1, 2, 3, …, 14, 15)
	m_{figure}_dir_{position}_{i}	the mean value of figure (thumb, index, middle, ring, pinky) direction of (X axis, Y axis, Z axis) in 3D coordinated system with window i (0, 1, 2, 3, …, 14, 15)
fset_std	std_{figure}_pos_{position}_{i}	the standard deviation value of figure (thumb, index, middle, ring, pinky) position of (X axis, Y axis, Z axis) in 3D coordinated system with window i (0, 1, 2, 3, …, 14, 15)
	std_{figure}_dir_{position}_{i}	the standard deviation value of figure (thumb, index, middle, ring, pinky) direction of (X axis, Y axis, Z axis) in 3D coordinated system with window i (0, 1, 2, 3, …, 14, 15)
fset_dist	dist_{figure}_{i}	the distance value of figure (thumb, index, middle, ring, pinky) to palm in 3D coordinated system with window i (0, 1, 2, 3, …, 14, 15)
	dist_{figures}_{i}	the distance value between two figures (thumb and index, index and middle, middle and ring, ring and pinky) in 3D coordinated system with window i (0, 1, 2, 3, …, 14, 15)
	m_{figure}_dir_{position}_{i}	the mean value of figure (thumb, index, middle, ring, pinky) direction of (X axis, Y axis, Z axis) in 3D coordinated system with window i (0, 1, 2, 3, …, 14, 15)
fset_bones_mean	m_{figure}_{bones}_{position}_{i}	the mean value of figure (thumb, index, middle, ring, pinky) with bones variable (fingertips Position, fingertips direction, distal phalanges position, intermediate phalanges position) of position (X axis, Y axis, Z axis) in 3D coordinated system with window i (0, 1, 2, 3, …, 14, 15)

**Table 2:** Average classification results for different datasets.

Dataset	Accuracy	Precision	Recall	F1-Score
fset_mean	93.459 ± 5.496	93.954 ± 5.046	93.459 ± 5.496	93.443 ± 5.488
fset_std	69.068 ± 10.994	70.189 ± 10.475	69.084 ± 10.999	68.701 ± 11.296
fset_dist	88.399 ± 9.762	87.565 ± 14.205	88.415 ± 9.776	88.211 ± 10.156
fset_bones_mean	93.610 ± 6.364	94.099 ± 5.800	93.610 ± 6.364	93.576 ± 6.387

**Table 3:** Average classification results for different machine learning models.

Machine Learning	Accuracy	Precision	Recall	F1-Score
LR	77.589 ± 13.268	78.686 ± 13.296	77.589 ± 13.268	77.489 ± 13.432
KNN	83.929 ± 14.965	84.823 ± 14.361	83.929 ± 14.965	83.812 ± 15.020
RF	88.943 ± 11.763	89.428 ± 11.438	88.943 ± 11.763	88.924 ± 11.769
ET	90.089 ± 11.336	90.423 ± 11.094	90.089 ± 11.336	90.044 ± 11.391
Hist-GB	89.018 ± 10.444	89.407 ± 10.171	89.018 ± 10.444	88.993 ± 10.444
LightGBM	88.795 ± 10.495	89.166 ± 10.265	88.795 ± 10.495	88.788 ± 10.460
ANN	88.938 ± 10.589	86.238 ± 18.598	89.009 ± 10.562	89.074 ± 10.523
1D-CNN	85.775 ± 13.319	87.008 ± 12.374	85.775 ± 13.319	85.648 ± 13.488
TabNet	82.129 ± 15.568	82.890 ± 15.116	82.129 ± 15.568	81.071 ± 16.696

**Table 4:** Average classification results for different feature selection methods.

Feature Selection	Accuracy	Precision	Recall	F1-Score
PCA	89.195 ± 9.863	89.643 ± 9.904	89.195 ± 9.863	88.990 ± 10.298
SFM	89.472 ± 10.369	90.023 ± 10.081	89.472 ± 10.369	89.383 ± 10.540
VAR	84.722 ± 12.089	85.403 ± 11.889	84.750 ± 12.062	84.474 ± 12.559
LASSO	79.850 ± 11.226	80.777 ± 10.999	79.850 ± 11.226	79.607 ± 11.425
ANN	89.861 ± 9.574	87.897 ± 16.680	89.861 ± 9.574	89.810 ± 9.701
TabNet	79.576 ± 20.161	80.626 ± 19.084	79.576 ± 20.161	79.440 ± 20.299
DML	90.260 ± 10.069	90.794 ± 9.942	90.288 ± 10.088	90.175 ± 10.365

**Table 5:** Average classification results for different feature selection methods and data.

Feature Selection	Dataset	Accuracy	Precision	Recall	F1-Score
PCA	fset_mean	95.833 ± 2.142	96.127 ± 1.918	95.833 ± 2.142	95.708 ± 2.376
	fset_std	74.351 ± 7.620	74.811 ± 8.326	74.351 ± 7.620	73.794 ± 8.828
	fset_dist	91.436 ± 3.914	92.125 ± 3.316	91.436 ± 3.914	91.347 ± 3.894
	fset_bones_mean	95.158 ± 2.480	95.508 ± 2.392	95.158 ± 2.480	95.112 ± 2.520
SFM	fset_mean	95.972 ± 2.214	96.309 ± 2.122	95.972 ± 2.214	95.962 ± 2.221
	fset_std	73.658 ± 6.126	74.626 ± 6.095	73.658 ± 6.126	73.380 ± 6.598
	fset_dist	91.824 ± 6.068	92.468 ± 5.854	91.824 ± 6.068	91.769 ± 6.067
	fset_bones_mean	96.436 ± 2.117	96.690 ± 1.893	96.436 ± 2.117	96.422 ± 2.125
VAR	fset_mean	91.759 ± 5.432	92.703 ± 4.326	91.759 ± 5.432	91.716 ± 5.444
	fset_std	72.316 ± 3.696	73.216 ± 3.646	72.427 ± 3.715	72.029 ± 4.175
	fset_dist	78.149 ± 12.181	78.756 ± 12.309	78.149 ± 12.181	77.497 ± 13.255
	fset_bones_mean	96.666 ± 1.924	96.939 ± 1.690	96.666 ± 1.924	96.656 ± 1.935
ANN	fset_mean	96.249 ± 1.757	96.592 ± 1.624	96.249 ± 1.757	96.353 ± 1.737
	fset_std	74.769 ± 3.531	75.768 ± 3.438	74.769 ± 3.531	74.539 ± 3.576
	fset_dist	92.037 ± 5.629	92.529 ± 5.368	92.037 ± 5.629	91.951 ± 5.763
	fset_bones_mean	96.390 ± 1.863	96.699 ± 1.658	96.390 ± 1.863	96.395 ± 1.857
DML	fset_mean	96.528 ± 2.240	96.801 ± 1.959	96.528 ± 2.240	96.532 ± 2.230
	fset_std	75.325 ± 5.210	76.196 ± 5.799	75.325 ± 5.210	74.910 ± 6.067
	fset_dist	92.362 ± 7.708	92.999 ± 7.754	92.473 ± 7.776	92.439 ± 7.818
	fset_bones_mean	96.824 ± 2.019	97.180 ± 1.726	96.824 ± 2.019	96.819 ± 2.029

**Table 6:** Average classification results for different feature selection methods and machine learning models.

Feature Selection	Machine Learning	Accuracy	Precision	Recall	F1-Score
PCA	RF	90.312 ± 7.808	90.702 ± 7.487	90.312 ± 7.808	90.258 ± 7.815
	ET	90.417 ± 8.265	91.024 ± 7.990	90.417 ± 8.265	90.350 ± 8.266
	Hist-GB	91.667 ± 7.586	91.834 ± 7.648	91.667 ± 7.586	91.605 ± 7.637
	ANN	91.083 ± 8.724	91.391 ± 8.660	91.083 ± 8.724	91.017 ± 8.791
SFM	RF	92.083 ± 8.705	92.366 ± 8.638	92.083 ± 8.705	92.081 ± 8.705
	ET	92.292 ± 9.549	92.536 ± 9.404	92.292 ± 9.549	92.262 ± 9.600
	Hist-GB	92.396 ± 7.213	92.609 ± 7.145	92.396 ± 7.213	92.398 ± 7.198
	ANN	92.438 ± 7.182	92.907 ± 6.697	92.438 ± 7.182	92.675 ± 6.734
VAR	RF	86.771 ± 12.002	87.114 ± 11.925	86.771 ± 12.002	86.700 ± 12.100
	ET	89.792 ± 9.476	90.028 ± 9.467	89.792 ± 9.476	89.784 ± 9.486
	Hist-GB	86.771 ± 9.796	87.075 ± 9.701	86.771 ± 9.796	86.729 ± 9.794
	ANN	87.188 ± 9.778	87.765 ± 9.229	87.438 ± 9.405	87.456 ± 9.367
ANN	RF	90.729 ± 11.526	91.149 ± 11.214	90.729 ± 11.526	90.810 ± 11.401
	ET	92.708 ± 9.079	92.896 ± 9.018	92.708 ± 9.079	92.720 ± 9.055
	Hist-GB	91.042 ± 9.531	91.314 ± 9.350	91.042 ± 9.531	91.060 ± 9.492
	ANN	90.000 ± 10.108	90.547 ± 10.148	90.000 ± 10.108	90.269 ± 10.205
DML	RF	93.125 ± 8.114	93.545 ± 7.726	93.125 ± 8.114	93.180 ± 8.002
	ET	94.062 ± 7.403	94.405 ± 7.017	94.062 ± 7.403	94.084 ± 7.376
	Hist-GB	92.812 ± 7.674	93.259 ± 7.300	92.812 ± 7.674	92.856 ± 7.612
	ANN	92.333 ± 8.331	93.218 ± 7.819	92.333 ± 8.331	92.647 ± 8.374

## Data Availability

The data presented in this work are available on request from the corresponding author.

## Abbreviations

The following abbreviations are used in this work:

HCI	Human–Computer Interaction
IoT	Internet of Things
LMC	Leap Motion Controller
DML	Double Machine Learning
SHAP	Shapley Additive Explanations
VAR	Variance Threshold
SFM	Select From Model
PCA	Principal Component Analysis
LASSO	Least Absolute Shrinkage and Selection Operator
ANN	Artificial Neural Network
CATE	Conditional Average Treatment Effect
LR	Logistic Regression
KNN	K-Nearest Neighbors
RF	Random Forest
ET	Extra Trees
Hist-GB	Histogram–based Gradient Boosting
LightGBM	Light Gradient Boosting Machine
1D-CNN	1-Dimensional Convolutional Neural Network
